# Oxygen Tension Is a Determinant of the Matrix-Forming Phenotype of Cultured Human Meniscal Fibrochondrocytes

**DOI:** 10.1371/journal.pone.0039339

**Published:** 2012-06-15

**Authors:** Adetola B. Adesida, Aillette Mulet-Sierra, Leila Laouar, Nadr M. Jomha

**Affiliations:** Department of Surgery, University of Alberta, Edmonton, Alberta, Canada; University of Rochester, United States of America

## Abstract

**Background:**

Meniscal cartilage displays a poor repair capacity, especially when injury is located in the avascular region of the tissue. Cell-based tissue engineering strategies to generate functional meniscus substitutes is a promising approach to treat meniscus injuries. Meniscus fibrochondrocytes (MFC) can be used in this approach. However, MFC are unable to retain their phenotype when expanded in culture. In this study, we explored the effect of oxygen tension on MFC expansion and on their matrix-forming phenotype.

**Methodology/Principal Findings:**

MFC were isolated from human menisci followed by basic fibroblast growth factor (FGF-2) mediated cell expansion in monolayer culture under normoxia (21%O_2_) or hypoxia (3%O_2_). Normoxia and hypoxia expanded MFC were seeded on to a collagen scaffold. The MFC seeded scaffolds (constructs) were cultured in a serum free chondrogenic medium for 3 weeks under normoxia and hypoxia. Constructs containing normoxia-expanded MFC were subsequently cultured under normoxia while those formed from hypoxia-expanded MFC were subsequently cultured under hypoxia. After 3 weeks of *in vitro* culture, the constructs were assessed biochemically, histologically and for gene expression via real-time reverse transcription-PCR assays. The results showed that constructs under normoxia produced a matrix with enhanced mRNA ratio (3.5-fold higher; p<0.001) of collagen type II to I. This was confirmed by enhanced deposition of collagen II using immuno-histochemistry. Furthermore, the constructs under hypoxia produced a matrix with higher mRNA ratio of aggrecan to versican (3.5-fold, p<0.05). However, both constructs had the same capacity to produce a glycosaminoglycan (GAG) –specific extracellular matrix.

**Conclusions:**

Our data provide evidence that oxygen tension is a key player in determining the matrix phenotype of cultured MFC. These findings suggest that the use of normal and low oxygen tension during MFC expansion and subsequent neo-tissue formation cultures may be important in engineering different regions of the meniscus.

## Introduction

The meniscus is a specialized fibrocartilaginous tissue located in the knee joint where it functions to aid joint stability, protect articular cartilage, absorb shock and transmit load [1]. The capacity to perform these functions is by virtue of its extracellular matrix (ECM) composition and assembly, which is accomplished entirely by meniscus fibrochondrocytes (MFC) [2], [3]. The ECM predominantly consists of type I collagen throughout, type II collagen in the inner meniscus, and proteoglycans of which aggrecan is predominant [4]–[6]. The meniscus shape and anatomy is also important [1], [2], [6]–[9]. Unfortunately, the reparative capacity of the meniscus is limited to the peripheral outer one-third while the avascular inner two-thirds does not heal [10]–[12]. Injuries located in the avascular region of the meniscus are often treated by partial or total menisectomy that can result in a high incidence of early OA [8], [13], [14]. Other modes of treatment include transplantation of meniscus allograft [15], [16], and implantation of biomaterial-based substitutes [17]–[21]. However, meniscus allograft suffers from donor shortage and risk of transmission of infectious diseases, while implantation of current biomaterial-based substitutes is only compatible with partial meniscectomy. To address these limitations, cell-based tissue engineering strategies to repair or to produce functional meniscus substitutes are of interest [22]–[27]. These approaches require an ideal biomaterial (scaffold) to support sufficient number of cells that exhibit the phenotypic characteristics of MFC.

While the ideal biomaterial and optimal cell population for cell-based tissue engineering of meniscus is yet to be identified, Arnoczky [28] has summarized what the important features of such a biomaterial should be, as well as several biological considerations [27], in creating a tissue engineered meniscus. These features include support of cell proliferation and ECM production, nutrient diffusion, reservoir of cytokines and biomechanical factors. The latter has also been addressed by Setton *et al* [29]. Although Arnoczky noted that MFC, fibroblasts and synovial cells have been used in conjunction with collagen and meniscal allograft based scaffolds to produce fibrocartilaginous tissues, additional studies were needed to characterize the cellular features and ECM produced. To this end several studies using a variety of: scaffolds, cell types and cytokine/growth factors combinations have been performed [20], [30]–[35].

In the present study, we focus on using MFC and a collagen sponge scaffold for three-dimensional (3D) culture of expanded human MFC. Nakata et al [30] and Gruber et al [35] have used bovine collagen sponges to culture monolayer expanded human MFC. However, monolayer culture of MFC results in de-differentiation (loss of matrix-forming phenotype) that is similar to articular chondrocyte de-differentiation, as evidenced by morphological changes to predominantly fibroblast-like cells, down-regulation of collagen type II expression and concomitant increase in collagen type I expression [30], [36]. In addition to these changes is the inability of the MFC to synthesize proteoglycan-rich ECM and form meniscus-like tissue [36].

In our previous work, we demonstrated that the addition of basic fibroblast growth factor (FGF-2) during monolayer expansion of human MFC mitigated the loss of their matrix-forming capacity [36]. In addition, we demonstrated that low oxygen tension further augmented their matrix-forming capacity [36].

In this study, we explored the effect of oxygen tension on the matrix-forming phenotype of cultured MFC. Our primary interest was to determine the effect of oxygen tension throughout MFC expansion and 3D culture on MFC's matrix-forming characteristics. We hypothesize that MFC expanded and chondrogenically stimulated under low oxygen tension culture will be more chondrogenic than their counterparts under normal oxygen tension.

## Materials and Methods

### Ethics Statement

Menisci were obtained from patients who underwent total knee replacement surgery. Local ethical committee approval of the University of Alberta, Edmonton, Canada was obtained for this study and institutional safety and ethical guidelines were followed. Ethics committee waived the need for written informed consent of patients as specimens used in the study were intended for discard in the normal course of surgical procedure. Extensive precautions were taken to preserve the privacy of the participants donating specimens.

### Isolation and expansion of human meniscus fibrochondrocytes (MFC)

Menisci were harvested from the knee joint of 3 female donors (mean age: 63.3±0.6 years [age 63–64 years]) undergoing total knee replacement surgery. MFCs were released by incubation for 16 hours at 37°C in type II collagenase (0.2% w/v; Worthington, Lakewood, NJ, USA) in a standard medium, high glucose Dulbecco's modified Eagle's medium (DMEM) 4.5 mg/ml D-Glucose supplemented with 5% FBS, 100 units/ml penicillin and 100 units/ml streptomycin, with L-glutamine (2 mM) (Invitrogen, Mississauga, Ontario, Canada) as described previously [36]. Isolated cells were plated at 10^4^ cells/cm^2^ and cultured in standard medium with FGF-2 (5 ng/ml; Humanzyme-Medicorp Inc., Montreal, Quebec, Canada) at 37°C under 3%O_2_ or 21%O_2_ in a humidified incubator with 5%CO_2_. After 3 weeks, when cells were sub-confluent, first passage cells (P1) cells were detached with trypsin-EDTA (Invitrogen) and counted. MFCs were then split at a 1∶2 ratio and culture was continued for 7 days to produce second passage (P2) cells. Doubling times of MFCs at P1 and P2 during the exponential growth phase was calculated as the slope of *T* against ln (*N*/*N*_0_), where *N*_0_ and *N* are the cell number at the beginning and end of the exponential growth time (*T*), respectively [37].

### DuraGen® Construct Cultures

DuraGen® collagen matrix (Integra Lifesciences, PlainsBoro, NJ, USA; 10 cm×12.5 cm; ∼3.5 mm total thickness collagen sponge with pore size of 115±20 µm) was cut into 6 mm diameter disks using a sterile biopsy punch. In order to limit variability between batches of DuraGen®, disks taken from the same batch of DuraGen® was used. The disks were placed in a 24-well plate and were carefully seeded on the collagen scaffold via a micropipette with 1×10^6^ MFC suspended in 10 µl of a defined serum-free chondrogenic medium consisting of high glucose DMEM containing 0.1 mM non-essential amino acids, 1 mM sodium pyruvate, 100 mM HEPES buffer, 1 mM sodium pyruvate, 100 U/ml penicillin, 100 µg/ml streptomycin, 0.29 mg/ml L-glutamine (Invitrogen) supplemented with 0.1 mM ascorbic acid 2-phosphate, 40 µg/ml L-proline, 10^−5^ M dexamethasone, 1× ITS+1 premix (Sigma-Aldrich, Oakville, Canada), 10 ng/ml TGF-β3 (Humanzyme-Medicorp Inc.). The seeded disks were transferred to a humidified incubator at 37°C with 21% O_2_ and 5% CO_2_ for 15 min to allow initial cell attachment. Thereafter, 100 µl of chondrogenic medium was gently added to the base of each well containing cell-seeded disks followed by a further 30 min incubation in a humidified incubator at 37°C with 21% O_2_ and 5% CO_2_. After the 30 min period, 1 ml of chondrogenic medium was added slowly to the base of each well until the entire seeded scaffolds were covered. The scaffolds with MFCs expanded under low oxygen tension (3% O_2_) were cultured for 3 weeks under 3% O_2_, and those seeded with cells expanded under 21% O_2_ were cultured for 3 weeks under 21% O_2_. Chondrogenic media exchange was performed twice per week. Thereafter, the scaffolds were processed biochemically for glycosaminoglycan (GAG) and DNA contents, histologically and immuno-histochemically for cartilage specific matrix proteins and at the molecular level by real time quantitative reverse transcription polymerase chain reaction (qRT-PCR) for gene expression analysis.

### Biochemical analysis

After culture, scaffolds were rinsed in phosphate buffer saline (PBS; Invitrogen), frozen overnight at −80°C and digested in proteinase K (1 mg/ml in 50 mM Tris with 1 mM EDTA, 1 mM iodoacetamide and 10 mg/ml pepstatin A – all from Sigma-Aldrich) for 16 h at 56°C. The sulphated GAG content was measured by 1,9 dimethylmethylene blue binding (Sigma-Aldrich) using chondroitin sulphate (Sigma-Aldrich) as standard [38]. The DNA content was determined using the CyQuant cell proliferation assay kit (Invitrogen) with supplied bacteriophage λ DNA as standard.

### Histology and Immuno-histochemistry

Tissues generated from the scaffold-cell cultures were fixed in 4% phosphate buffered formalin, processed into paraffin wax, sectioned at 5 µm and stained with 1% alcian blue and counterstained with 1% neutral red stain, to reveal sulphated proteoglycan (GAG) matrix depositions. Other sections were probed with antibodies raised against collagen types I and II. Sections were pretreated with 0.1% w/v trypsin and then incubated with antibodies against collagen I (MAB3391; at 1∶100 dilution) from Millipore, Temecula, California, USA, or collagen II (II-II6B3; at 1∶50 dilution) from Developmental Studies Hybridoma Bank at University of Iowa, USA. Immuno-localised antigens were visualized with goat anti-mouse IgG biotinylated secondary antibody (Dako Canada Inc, Mississauga, Ontario, Canada) and a streptavidin-horseradish peroxidase labeling kit with 3,3′-diaminobenzidine (Dako). Images were captured using an Omano OM159T biological trinocular microscope (Microscope Store, Virginia, USA) fitted with an Optixcam summit series 5 MP digital camera, Optixcam software and assembled in Adobe Photoshop (Adobe Systems Inc. San Jose, USA).

### Gene Expression Analysis

Total RNA was extracted from the monolayer cultures of MFCs and cell-scaffold constructs using Trizol (Invitrogen) after grinding with Molecular Grinding Resin (Geno Technology Inc. St Louis, USA) in combination with the use of Aurum Total RNA Fatty and Fibrous Tissue Kit (Bio-Rad, Mississauga, Ontario, Canada) and after removal of contaminating genomic DNA from the pellets by DNase treatment. To minimize changes in gene expression, monolayer cultures of MFCs and cell-scaffold constructs were immediately (less than 1 minute) transferred into Trizol. Total RNA (100 ng) in a 40 µl reaction was reverse transcribed to cDNA using GoScript reverse transcriptase (Fisher Scientific, Whitby, Ontario, Canada) primed with random primers oligonucleotides. qRT-PCR was performed in a DNA Engine Opticon I Continuous Fluorescence Detection System (Bio-Rad) using hot start Taq and SYBR Green detection (Eurogentec North America Inc, San Diego, CA, USA). Primer sequences (Table 1) were taken from previously published work [36], [39], [40]. All primers were obtained from Invitrogen, Mississauga, Ontario, Canada. Gene (mRNA) expression levels for each primer set were normalized to the expression level of human RNA polymerase II (RPII) [41], by the 2−^Δct^ method [42].

**Table 1:** Primer sequences used in quantitative real-time PCR (all primers were purchased from Invitrogen, Mississauga, Ontario, Canada).

Gene	Primer sequence	Direction	Reference.
Aggrecan	5′AGGGCGAGTGGAATGATGTT3′ 5′GGTGGCTGTGCCCTTTTTAC3′	(Fwd) (Rev)	[36]
Biglycan	5′TTGCCCCCAAACCTGTACTG3′ 5′AAAACCGGTGTCTGGGACTCT3′	(Fwd) (Rev)	[36]
Collagen 1A2	5′TTGCCCAAAGTTGTCCTCTTCT3′ 5′AGCTTCTGTGGAACCATGGAA3′	(Fwd) (Rev)	[36], [40]
Collagen 2A1	5′CTGCAAAATAAAATCTCGGTGTTCT3′ 5′GGGCATTTGACTCACACCAGT3′	(Fwd) (Rev)	[36], [40]
Collagen 2B	5′CTGCTCGTCGCCGCTGTCCTT3′ 5′AAGGGTCCCAGGTTCTCCATC3′	(Fwd) (Rev)	[39]
Decorin	5′CAAGCTTAATTGTTAATATTCCCTAAACAC3′ 5′ATTTTATGAAGGGAGAAGACATTGGTTTGTTGACA3′	(Fwd) (Rev)	[36]
RPII	5′GACACAGGACCACTCATGAAGT3′ 5′GTGCGGCTGCTTCCATAAG3′	(Fwd) (Rev)	[41]
SOX9	5′CTTTGGTTTGTGTTCGTGTTTTG3′ 5′AGAGAAAGAAAAAGGGAAAGGTAAGTTT3′	(Fwd) (Rev)	[36]
Versican	5′TGGAATGATGTTCCCTGCAA3′ 5′AAGGTCTTGGCATTTTCTACAACAG3′	(Fwd) (Rev)	[39]

### Statistical analysis

A total of three independent experiments were performed with a minimum repeat of triplicates. Data are presented as mean ± standard deviation of at least 3 replicates of measurements acquired from 3 donor specimens. Statistical analyses were performed using SPSS (version 18). Statistical differences among two or multiple study groups were assessed by paired two-tailed distribution Student's *t*-test or by one-way ANOVA with Tukey's multiple comparison post-tests. All statistical differences were considered to be significant with *p*<0.05.

## Results

### Cell culture and biochemical analysis

**Figure 1:**
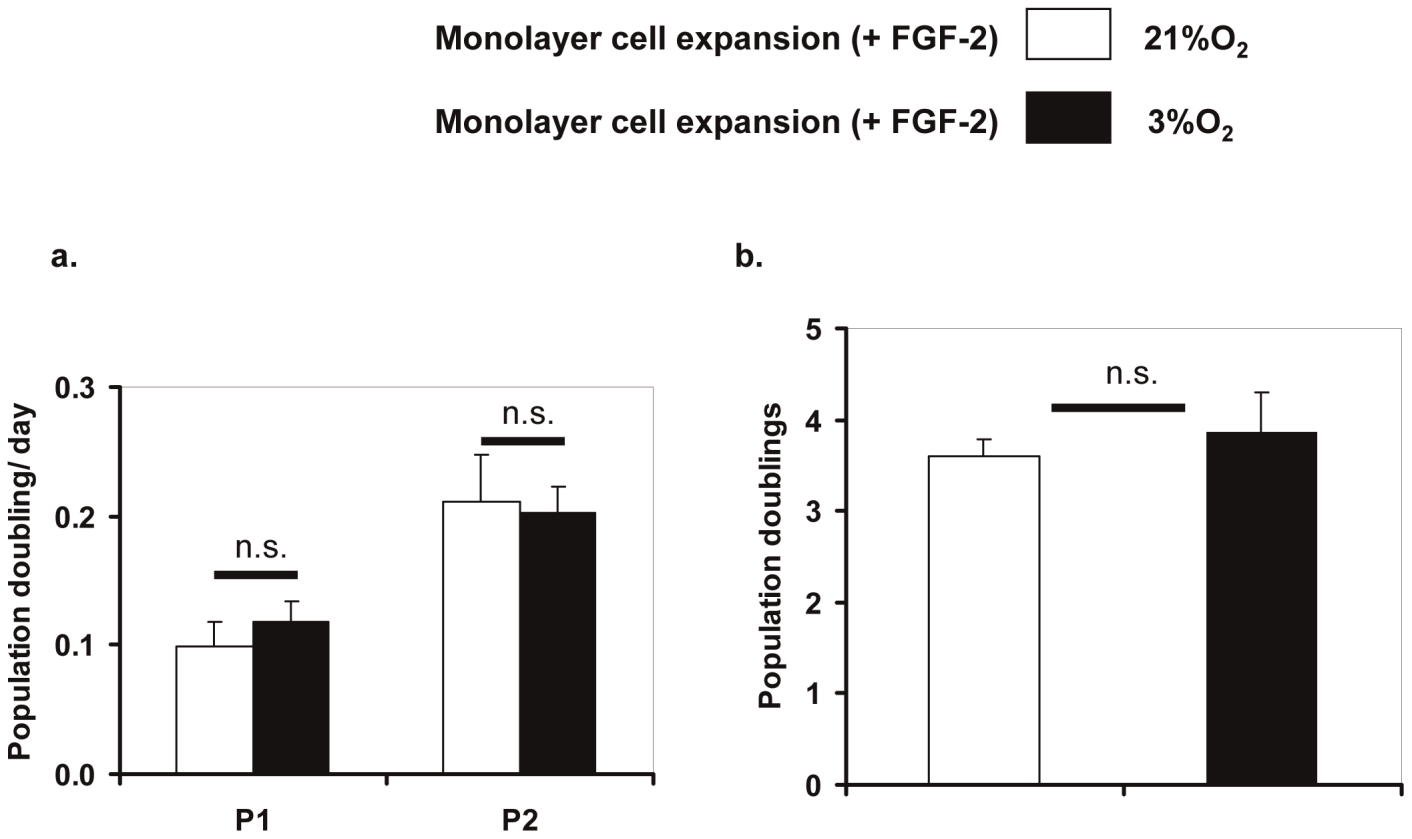
Proliferation rate of human meniscus fibrochondroytes under normal and low oxygen tension in the presence of FGF-2. a) Mean cell doubling rate for P1 and P2 human meniscus fibrochondrocytes from 3 donors in the presence of FGF-2. b) Mean total population doubling of P2 human meniscus fibrochondrocytes. Data is expressed as mean ± SD of 3 donors (n = 3, N = 3). FGF-2, fibroblast growth factor 2; P1, passage 1; P2, passage 2. Student's t statistics; not significant (n.s.; *p*>0.05).

MFCs were isolated from human knee menisci and cultured in monolayer in the presence of FGF-2 under normal (21% O_2_) or low (3% O_2_) oxygen tension. The cells proliferated well with an elongated spindle-like morphology with rates of cell population doubling of: 0.1±0.02 per day at P1 and 0.21±0.04 per day at P2 under normal oxygen tension, and at population doubling rate of: 0.12±0.02 per day at P1 and 0.2±0.02 per day at P2 under low oxygen tension. There was no significant difference (*p*>0.05; *p* = 0.21 at P1 and *p* = 0.80 at P2) between cell population doubling rates under normal and low oxygen tension culture conditions (Fig. 1a). The mean total cell population doublings (i.e. P2 cells) prior to cell seeding on collagen scaffold was 3.61±0.18, for cells cultured under normal oxygen tension and 3.87±0.43 for cells cultured under low oxygen tension. There was no significant difference (*p* = 0.54) between the mean total population doublings at normal and low oxygen tension (Fig. 1b).

### Histology

**Figure 2:**
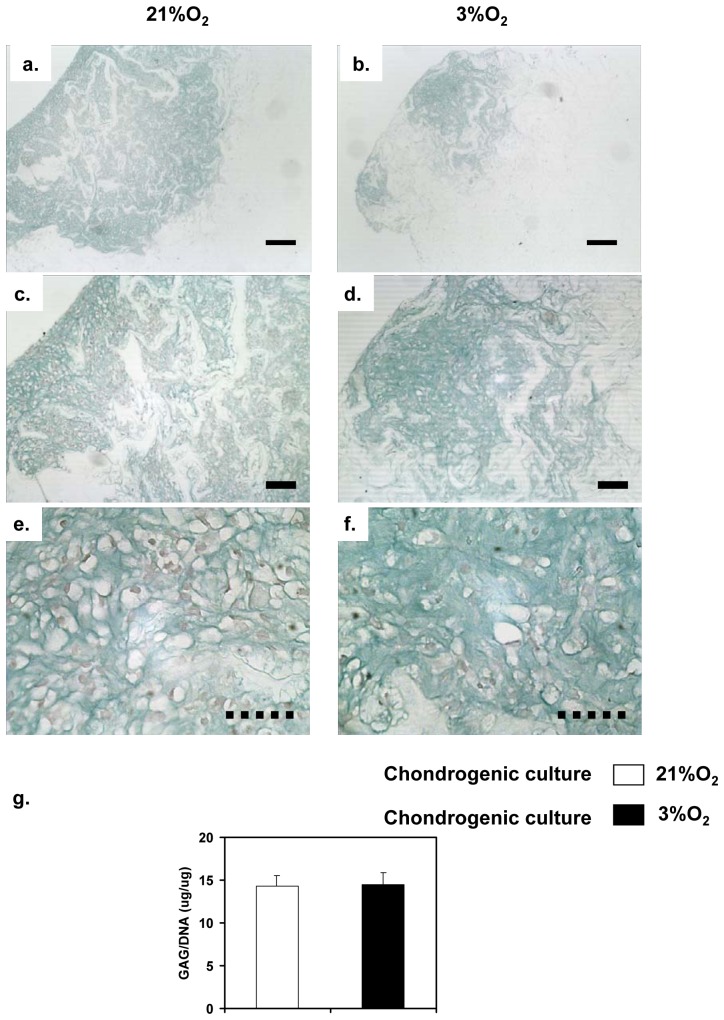
Histological and biochemical analysis of Duragen®-meniscus fibrochondrocytes (MFCs) constructs after culture for 21 days under normoxic (21% O_2_) and hypoxic (3%O_2_) conditions. a) Low (4×); c) medium (10×); e) high (40×) magnification photomicrographs of analysis of paraffin wax embedded sections (5 µm thickness) stained with alcian blue/neutral red stain (for sulphated GAG detection). Constructs were seeded with normoxia-expanded MFCs and were cultured under normoxia. b) Low (4×); d) medium (10×); f) high (40×) power magnification photomicrographs of alcian blue/neutral red (for sulphated GAG) stained constructs seeded with hypoxia-expanded MFCs and were cultured under hypoxia. Solid scale bar = 100 µm and dotted bar = 50 µm. (g) Biochemical analysis was used to evaluate the glycosaminoglycan (GAG) and DNA contents of constructs after 21 days culture in serum-free chondrogenic medium under normal (21%O_2_) and low oxygen tension (3%O_2_). Data is presented here as chondrogenic capacity (i.e. GAG levels normalized to DNA content) of constructs generated from 3 independent donors and it represents the mean ± SD (n = 10, N = 3). Student's t statistics; not significant (n.s.; *p*>0.05).

MFC-seeded Duragen® scaffold constructs were cultured in serum-free chondrogenic medium. Constructs generated from cells expanded under normal oxygen tension were subsequently cultured under same oxygen tension and constructs formed from hypoxia-expanded meniscus cells were subsequently cultured under the same low oxygen tension. Histological assessment of the constructs after 3 weeks of cultivation using Alcian blue staining for sulphated proteoglycan showed that there was positive deposition of proteoglycan (GAG)-rich matrix as indicated by greenish-blue color, and good migration of meniscus cells into the porous Duragen® collagen scaffold (Figs. 2a and 2c – normoxic conditions and Figs. 2b and 2d – hypoxic conditions). Furthermore, the majority of cells within the GAG-rich matrix had a rounded chondrocyte-like morphology as seen at higher magnifications (Figs. 2e – normoxic conditions and 2f – hypoxic conditions). Quantitative assessment of total GAG per cellular DNA content in the constructs showed no statistical significance (*p*>0.05) between constructs cultivated under normal and low oxygen tensions (Fig. 2g). Interestingly, there were negligible differences in the ratio of GAG/DNA amongst the different donors examined in this study. The ratios for the three donors ranged from 11.25–16.28 for constructs cultivated under normal oxygen tension and 12.41–16.84 for constructs cultured under low oxygen tension.

### Gene expression analysis

**Figure 3:**
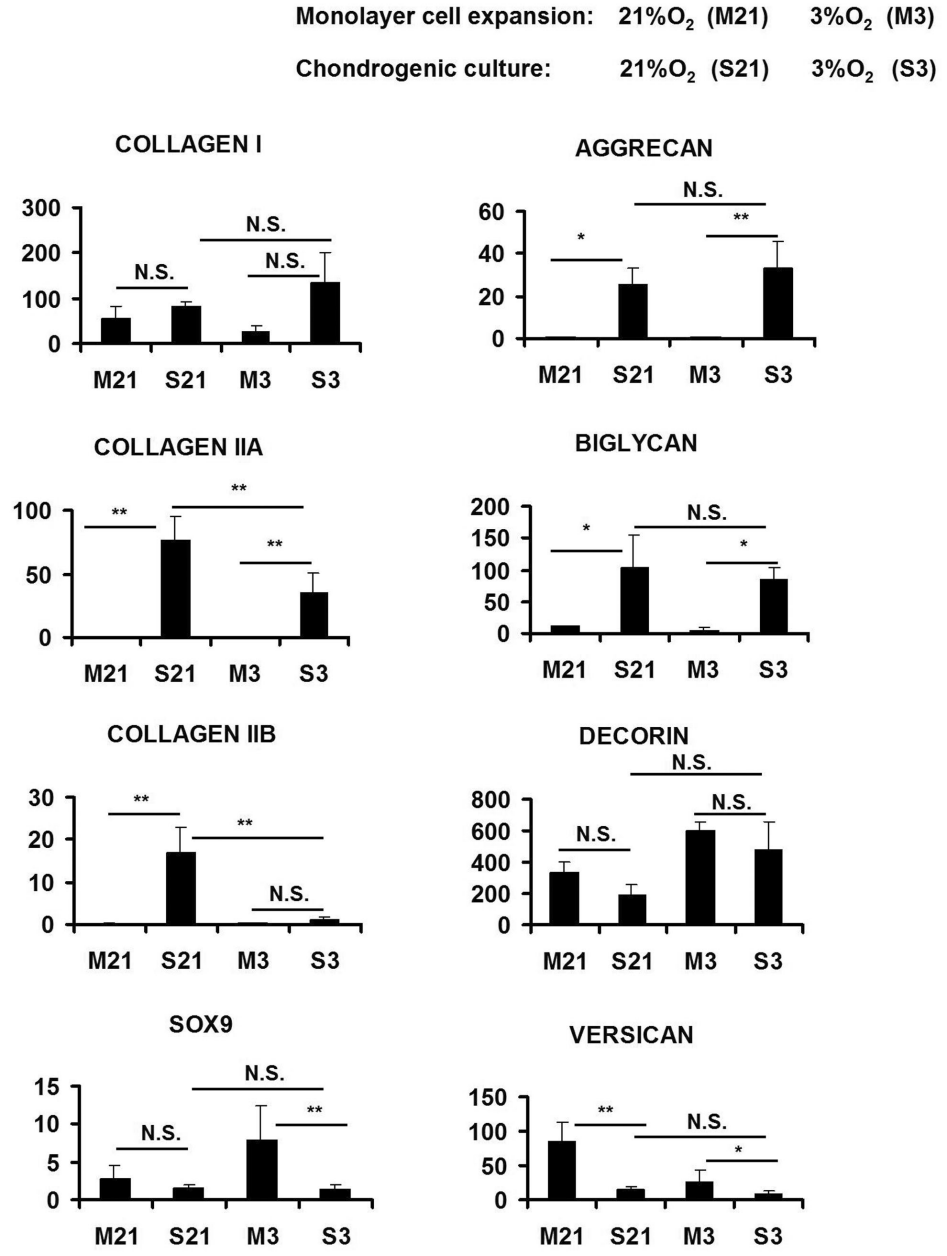
Gene expression profile of Duragen®-meniscus fibrochondrocytes constructs cultured for 21 days under normoxic (21%O_2_) and hypoxic (3%O_2_) conditions. The effect of oxygen tension on the matrix-forming capacity of differentially expanded MFCs prior to (monolayer cell culture; M) and after seeding and culture on Duragen® collagen scaffold (S) was investigated by real-time quantitative reverse-transcription-polymerase chain reaction (qRT-PCR). Total RNA isolated from monolayer cells culture (M) and cells-scaffold constructs (S) were analyzed for the mRNA expression of: the transcription factor, *Sox9*, and members of the collagen gene family and sulphated proteoglycans found in the extracellular matrix of meniscal fibrocartilage. All *y*-axes represent relative gene expression levels normalized to human RNA polymerase II (RPII). Data represents mean ± SD; one-way ANOVA with Tukey's post hoc test: n.s. (not significant), * = *p*<0.05, ** = *p*<0.001, n = 10, N = 3.

In order to further characterize the ECM formed in the generated constructs, we examined gene expression in all constructs using quantitative real-time RT-PCR and in monolayer cell cultures prior to seeding and culture on scaffolds (Fig. 3). The expression of collagen I (*Col1a2* transcript) was not statistically significant (*p* = 0.95) between constructs cultured under low and normal oxygen tensions as well as between monolayer cell cultures and cell-scaffold constructs. The expression of *Col1a2* in monolayer MFC culture under normal oxygen tension was 2.3 fold higher than its expression in monolayer cultures of MFC under low oxygen tension. However, the fold difference was not statistically significant (p = 0.896; not shown in Fig. 3).

The early developmental form of type II collagen gene, *Col2a1*, was expressed 2.14-fold (*p* = 0.03) higher in constructs cultivated under 21%O_2_ relative to expression in constructs cultured under 3% O_2_. The expression of *Col2a1* in monolayer cell culture compared to its expression in constructs under normal oxygen tension was 5×10^6^ times less (*p*<0.0001) –Fig. 3. Under low oxygen tension, monolayer expression of *Col2a1* was over 580,000 times less than its expression in constructs. The expression of *Col2a1* in monolayer cultures under normal and low oxygen tension was not statistically significant between the different donors but the relative mean expression was 4 fold higher under low oxygen tension (not shown in Fig. 3).

**Figure 4:**
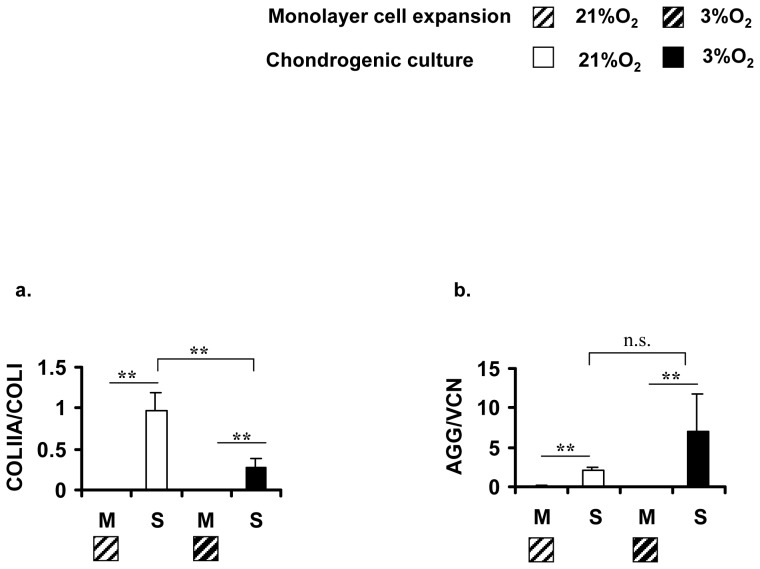
Ratios of mRNA expression levels of collagen type II (*Col2a1*) to collagen type I (*Col1a2*) and of aggrecan (AGG) to versican (VCN). Data represents mean ± SD; one-way ANOVA with Tukey's post hoc test: n.s. (not significant), * = *p*<0.05, ** = *p*<0.001, n = 10, N = 3.

The transcript expression level of the mature isoform of type II collagen, *Col2b*, was consistently elevated with statistical significance (17-fold; *p* = 0.00005) in all constructs formed under normal oxygen tension compared to constructs from low oxygen tension. The expression of *Col2b* in monolayer cultures under normal oxygen tension was significantly down regulated by over a 100-fold relative to its expression in constructs under the same oxygen tension. However, there was no difference between the expression of *Col2b*, in monolayer cultures and in constructs under low oxygen tension. mRNA ratio of type II collagen (*Col2a1*) to collagen type I (*Col1a2*) as a parameter for chondrogenic induction, was 3.5-fold higher with statistical significance (*p* = 0.0008) in constructs cultured under normal oxygen tension relative to constructs cultured under low oxygen tension (Fig. 4a). An enhancement in *Sox9* expression did not accompany the increased type II collagen expression in constructs cultured under normal oxygen tension. *Sox9* expression level was similar in constructs regardless of the oxygen tension during culture (Fig. 3). Furthermore, there was no difference in *Sox9* expression between monolayer culture and constructs at normal oxygen tension. In contrast, there was a significant 6-fold down-regulation in constructs under low oxygen tension relative to expression in monolayer cell culture prior to seeding for cell-scaffold constructs. Sox9 expression was 3-fold higher (*p* = 0.04) in monolayer cultures under low oxygen tension than in monolayer culture under normal oxygen (Fig. 3).

The mRNA expression of a panel of proteoglycans expressed in knee meniscus including aggrecan, biglycan, decorin and versican was examined in monolayer cell and cell-scaffold constructs. With the exception of versican, the remaining proteoglycans were not differentially expressed between the constructs cultured under normal and low oxygen tension (Fig. 3). The mRNA expression level of versican was 2-fold higher in constructs cultivated under normal oxygen tension relative those cultured under low oxygen tension (Fig. 3). mRNA ratio of aggrecan to versican (i.e. AGG/VCN, another index of chondrogenic induction) was 3.5-fold higher with statistical significance (*p* = 0.049) in constructs cultured under 3% O_2_ relative to those cultured under normal oxygen tension (Fig. 4b). The expression of aggrecan and biglycan in monolayer cultures of MFCs was significantly lowered relative to their expression in cell-scaffold constructs. While the expression level of decorin was not different between MFCs in monolayer and chondrogenically stimulated cell-scaffold constructs regardless of the oxygen tension. In contrast, versican's expression was significantly higher in monolayer MFCs than in MFC-scaffold constructs by 5- and 4-fold, under normal and low oxygen tension, respectively.

### Immunohistochemistry

**Figure 5:**
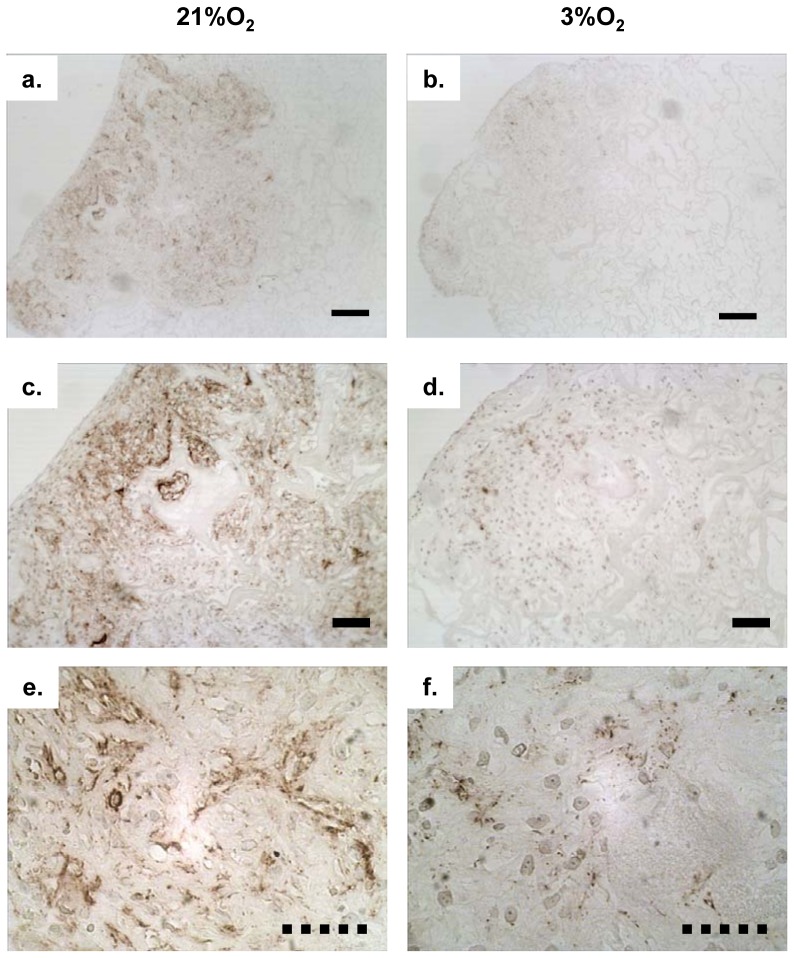
Immunohistochemistry (IHC) of extracellular matrix deposition of collagen type II : Collagen type II as indicated by brown staining was detected in Duragen®-meniscus fibrochondrocytes constructs cultured for 21 days under normoxic (21%O_2_) and hypoxic (3%O_2_) conditions using paraffin-wax embedded sections (5 µm thickness). a) Low (4×); c) medium (10×); e) high (40×) magnification of IHC photomicrographs of collagen II stained sections of constructs containing normoxia expanded MFCs followed by culture under normoxia. b) Low (4×); d) medium (10×); f) high (40×) magnification of IHC photomicrographs of collagen II stained sections of constructs containing hypoxia expanded MFCs followed by culture under hypoxic conditions.

Immuno-histochemical assessment of the constructs with collagen II antibody revealed a more intense (brown stain) staining of collagen II in constructs generated from normal oxygen tension (Figs. 5a, 5c and 5e) compared to constructs from low oxygen tension (Figs. 5b, 5d and 5f), supporting the enhanced mRNA expression of type II collagen in constructs cultivated under normal oxygen tension (Fig. 4a). We also stained our constructs for type I collagen, however, MFC-free collagen scaffold (empty control scaffolds) cross-reacted with the collagen I antibody, therefore the data was excluded from this study.

## Discussion

Cells lose their multiplication and phenotypic characteristics when they are serially sub-cultivated. However, both of these characteristics are critical to obtain sufficient cell numbers for the translation of tissue engineering strategies into cell-based therapeutics. Thus, identifying the environmental conditions that minimizes the loss of the matrix-forming phenotype of MFC is important for the generation of functional cell-based meniscus substitutes.

In this study, we explored the effect of oxygen tension on MFC proliferation and subsequent chondrogenic matrix formation on a collagen I-based scaffold. Two experimental groups were defined as normal and low oxygen tensions. While we recognize that the meniscus is distinct from cartilage, we have focused on re-expression of the chondrocyte-like phenotype of MFCs in this study, since like chondrocytes, MFCs undergo the loss of collagen II and aggrecan expression in monolayer cell culture. Thus, we assume that the re-expression of these chondrogenic markers in tissue engineered meniscus constructs may serve as an indicator of restoration of the functional matrix-forming phenotype of cultured MFCs.

Both experimental groups (normal and low oxygen tension) produced the same amount of GAG-rich matrix despite different oxygen tension. This finding is further corroborated by the gene expression of a panel of GAG-specific matrix proteins (aggrecan, biglycan, decorin and versican) in Figure 3, which demonstrated that the transcript expression of the proteins was independent of the oxygen tension during chondrogenic culture of the cell-scaffold constructs. The oxygen tension was maintained at the same level during both the multiplication stage and the subsequent chondrogenic culture stage to control for this variable.

Our study demonstrated that normoxia-expanded and subsequent chondrogenic cultured MFC deposited more collagen II protein within the constructs than did their hypoxia-expanded counterparts. These results did not support our hypothesis. The mechanism underlying our findings is unclear; however, it is reasonable to postulate that the two experimental MFC groups in this study responded positively to chondrogenic stimuli through differential aspects of the chondrogenic pathway. This assumption is based on the fact that regardless of the oxygen tension, the expression of chondrogenic markers (*Col2a1*, *Col2b* and aggrecan) in monolayer cell culture prior to chondrogenic stimulation on scaffolds was significantly lower compared to their expression in cell-scaffold constructs after chondrogenic stimulation. Furthermore, the gene expression ratio of *Col2a1*/*Col1a2*, as a chondrogenic index [43], was significantly higher in cell-scaffold constructs under normoxia culture conditions. Another observation was that the enhanced expression of collagen II in the constructs formed from normoxia-expanded and cultivated MFCs was not accompanied by an increased expression of *Sox9* (a transcription factor known to facilitate collagen II expression). While the lack of correlation between collagen II and *Sox9* is not unusual as per the findings of several studies [44]–[46], our data is consistent with reports that there are other factors that regulate collagen II transcription even though *Sox9* appears necessary for collagen II expression [44], [46]. Oxygen tension may be one of these regulatory factors.

In agreement with our previous study [36], under normoxic conditions, MFC proliferated in culture. However, in contrast and regardless of the oxygen tension, the proliferation rate of MFC at passage 2 was higher (∼2-fold) than their counterparts at passage 1 in this study. The reason for this discrepancy is not clear but may be related to differences in donor age [47], [48]. The donor age range in our previous study was 48–69 years, while in this study it was 63–64 years. In addition, there was no difference between MFCs proliferation rates under normal and low oxygen tensions. This suggested that the two MFC populations or experimental groups in this study were homogenous or, alternatively, were distinct but with similar proliferation rates.

Our findings may be a result of discriminatory selection of MFC within the distinct regions of the meniscus. Nakata *et al* [30] reported three distinguishable cell types in monolayer culture of MFC: elongated fibroblast-like cells, polygonal cells and small round chondrocyte-like cells, and Hellio Le Graverand *et al* [49] showed that rabbit MFC located in the inner avascular region resemble articular chondrocytes as they are rounded in morphology and well separated from one another by surrounding extracellular matrix. MFC in the outer region are spindle-shaped with numerous projections. In addition, Mauck et al [50] demonstrated that bovine MFCs from the outer vascular region displayed the most multi-lineage differentiation plasticity amongst the outer (vascular), inner (avascular) and horn (mixed) regions. Furthermore, we have previously shown that MFC from the outer meniscus are more responsive to chondrogenic and hypoxic stimuli than MFC from the inner meniscus based on transcript expression of collagen II and *Sox9* [40]. Thus, some results from our study can be explained in this manner. For example, the up-regulation of the collagen II and versican expression in normoxic culture conditions could be due to selective proliferation of those MFC from the vascular area that normally would be exposed to higher oxygen tensions. Conversely, the down-regulation of versican in the hypoxic conditions could be due to preferential proliferation and culture of those MFC in the avascular area, which would have natively resided in hypoxic microenvironment. While dedicated studies to confirm these possibilities merits attention, aspects of our data appear to support this explanation. For example, the mean expression of *col1a2* was higher under normal oxygen tension and the mean expression of *col2a1* was higher in monolayer cultures under low oxygen tension albeit with statistical indifference between donors. In our previous work, we showed that the expression of *col1a2* was higher in cells isolated from the outer vascular region of the meniscus, while the expression of *col2a1* was higher in cells from the inner avascular region. [40]. However, it is noteworthy that in another study, we demonstrated that MFCs' expanded under normal oxygen tension and subsequent chondrogenic culture under hypoxic conditions (5% O_2_) resulted in an enhanced GAG-specific matrix synthesis compared to MFCs cultured in 21% O_2_ [36]. Nonetheless, the data herein opens a new perspective that maintaining the same oxygen tension throughout MFC multiplication and subsequent chondrogenic culture plays a determinant role in MFC's matrix-forming phenotype, with the possibility that the mechanism of action is either by selective proliferation of MFC with different chondrogenic propensities or by activation of distinct aspects of the chondrogenic pathway.

Taken together, our results suggest that the proliferation rates of cultured MFC are equal regardless of oxygen tension investigated in this study. In addition, the expansion of MFCs under low oxygen tension prior to chondrogenic culture appears to offer no advantage over expansion and culture in normoxic conditions. Furthermore, the amount of GAG-specific matrix formed is independent of oxygen tension. However, the matrix-forming phenotype of MFC is dependent on the oxygen tension during MFC propagation and subsequent chondrogenic culture. The findings of this study have important implications in MFC-based tissue engineering repair strategies of the meniscus and may particularly be relevant in engineering specific regions of the meniscus. For example, normoxia-expanded MFC may be useful in engineering the inner meniscus, which is collagen II rich than the outer meniscus. However, a further study is required to ascertain whether selective expansion of cells from the inner or outer meniscus is the basis for our data.
